# Seven esophageal perforation cases after aortic replacement/stenting for thoracic aortic dissection or aneurysm

**DOI:** 10.1186/s40792-017-0354-7

**Published:** 2017-06-19

**Authors:** Yoshihisa Yaguchi, Yoshimasa Kumata, Masahiro Horikawa, Takashi Kiyokawa, Tsuyoshi Inaba, Ryoji Fukushima

**Affiliations:** Department of Surgery, Teikyo University School of Medicine, 2-11-1, Kaga, Itabashi-ku, Tokyo, 173-8605 Japan

**Keywords:** Esophageal perforation, Aortic dissection, Aortic aneurysm, Aortic replacement, TEVAR

## Abstract

**Background:**

Esophageal perforation after aortic replacement/stenting for aortic dissection or aneurysm is a rare but severe complication. However, its cause, standard treatment, and prognosis are unclear. We analyzed the treatment and outcome retrospectively from seven cases experienced at our hospital.

**Case presentation:**

The median age of the patients was 70 years (range, 41–86), and six of the seven cases were male. As the first treatment, aortic replacement techniques were performed in five, and thoracic endovascular aortic repair (TEVAR) procedure was performed in two. We evaluated the treatment of the perforation, the cause of death, and the median survival time after reparative surgery (esophagectomy).

Initial treatment of the perforation was esophagectomy without reconstruction in six and esophagogastric bypass (later, esophagectomy was performed) in one. Three of seven cases could be discharged from hospital or moved to another hospital, but two of these three cases died of major bleeding on postoperative days 320 and 645. The other four esophagectomy cases died in hospital because of sepsis on postoperative days 14, 30, and 41 and major bleeding on postoperative day 54. The one surviving case was a 65-year-old man who underwent reconstruction, and was still alive without signs of infection at 424 days postoperatively.

**Conclusion:**

The prognosis of esophageal perforation cases after aortic replacement/stenting for thoracic aortic dissection or aneurysm is poor, though there were some cases with relatively long survival. Therefore, the indication for invasive esophagectomy should be decided carefully. Control of infection including regional infection is essential for successful treatment.

## Background

Esophageal perforation after aortic replacement or stent grafting for aortic dissection or aneurysm is a rare but potentially fatal complication. However, its cause, standard treatment, and prognosis are unclear [1–3].

There are some reports with the key word of aorto-esophageal fistula (AEF) between the thoracic aorta and the esophagus. However, it is unclear whether the definition of AEF includes esophageal perforation after aortic replacement, as artificial grafts do not fistulize. In fact, most AEF-related papers are about cases after thoracic endovascular aortic repair (TEVAR) procedure [3–5].

The development of esophageal perforation indicates the occurrence and persistence of infection leading to mediastinitis or sepsis. However, it is interesting to consider the cause of the infection and to speculate on what happened at the site of the local lesion, that is, whether esophageal perforation is caused by localized infection including artificial graft infection or the infection is caused by esophageal perforation. In any case, perforation becomes the cause of continuous infection, and esophagectomy may be necessary for definitive treatment. However, esophagectomy is highly invasive surgery, and the indication should be carefully considered, particularly after major cardiovascular surgery. It is not also sure whether localized infection including graft/stent infection is improved after esophagectomy.

We retrospectively reviewed our seven cases of esophageal perforation after aortic replacement/stenting and analyzed their treatment and outcome.

## Case presentation

We experienced seven esophageal perforation cases after aortic replacement/stenting for thoracic aortic dissection or aneurysm from 2013 to 2015 (Table 1). The median age of patients was 70 years (range, 41–86), and six of the seven cases were male. The cardiovascular surgical procedures included two cases of total arch replacement for aortic dissection, two cases of replacement techniques, and two cases of endovascular aneurysm repair for aortic aneurysm. Valve-sparing aortic root replacement was initially performed for acute aortic dissection with Marfan syndrome case. Several operations were performed in patient nos. 3, 5, and 7 because of disease progression. Continuous localized infection after cardiovascular surgery was observed in patient nos. 2, 3, and 6. The symptoms leading to the diagnosis of eshophageal perforation and the period from cardiovascular surgical procedure to esophageal perforation are shown in Table 1. We evaluated the treatment of perforation, cause of death, and median survival time after reparative surgery (esophagectomy).

**Table 1:** Patient’s characteristics

No.	Age, sex	Aortic disease	Cardiovascular surgical procedure	Continuous localized infection after surgery	Symptoms leading to diagnosis of esophageal perforation	Period from surgical procedure to esophageal perforation
1	65, male	Acute aortic dissection	Total arch replacement	No	Fever	586 days
2	70, male	Aortic aneurysm	Total arch replacement	Yes	Saliva leakage into thoracic cavity	349 days
3	86, male	Rupture of aortic aneurysm	1. TEVAR 2. Aortic replacement (descending aorta)	Yes	Hematemesis	12 days
4	82, male	Acute aortic dissection	Total arch replacement	No	Fever	960 days
5	81, male	Aortic aneurysm	1. TEVAR 2. Aortic replacement (descending aorta)	No	None (intraoperative diagnosis of aortic replacement)	714 days
6	61, female	Infectious aortic aneurysm	Aortic replacement (descending aorta)	Yes	Back pain	101 days
7	41, male	Acute aortic dissection with Marfan syndrome	1. Valve-sparing aortic root replacement 2. Total arch replacement 3. TEVAR	No	None (follow-up CT)	2205 days

*TEVAR* thoracic endovascular aortic repair

Initial treatment of the perforation was esophagectomy without reconstruction in six and esophagogastric bypass (with later esophagectomy) in one. Gastrostomy for enteral alimentation was performed in all six esophagectomy cases. Aortic replacement of the descending aorta was performed at the same time in patient no. 3, and evacuation of hematoma in the left thoracic cavity caused by dissection was needed in patient no. 7. Median operation time was 375 min (range, 327–638 min), and blood loss was 500 ml (range, 85–2374 ml). Postoperative complications were observed in six of seven cases (Table 2).

**Table 2:** Treatment and prognosis

No.	Initial treatment of perforation	Operation time and blood loss	Complications	Discharge	Outcome of postoperative observation period	Cause of death
1	Esophagectomy without reconstruction, gastrostomy, and esophagostomy (left thoracotomy)	375 min 500 ml	SSI	Yes	Survival 424 days	–
2	Esophagectomy without reconstruction, gastrostomy, and esophagostomy (right thoracotomy)	345 min 251 ml	SSI	No	Death 54 days	Bleeding
3	Esophagectomy without reconstruction, gastrostomy, and esophagostomy with aortic replacement (descending aorta) (left thoracotomy)	514 min 3231 ml	Sepsis Mediastinitis	No	Death 41 days	Sepsis Mediastinitis
4	Esophagectomy without reconstruction, gastrostomy, and esophagostomy (right thoracotomy)	327 min 150 ml	Sepsis Myocardial infarction	No	Death 14 days	Sepsis Myocardial infarction
5	Esophagectomy without reconstruction, gastrostomy, and esophagostomy (right thoractomy)	638 min 3704 ml	Sepsis	No	Death 30 days	Sepsis Intrapulmonary hemorrhage
6	Esophagogastric bypass	326 min 85 ml	None	Yes	Death 645 days	Bleeding
7	Esophagectomy without reconstruction, gastrostomy, and esophagostomy with hematoma evacuation (right thoracotomy)	554 min 2374 ml	Empyema Pneumonitis Mediastinitis	Yes	Death 320 days	Bleeding

*SSI* surgical site infection

Patient nos. 1, 6, and 7 could be discharged from hospital or moved to another hospital, but patient nos. 6 and 7 died of major bleeding on postoperative days 320 and 645. These two cases experienced chronic regional infection of the artificial graft/stent. The other four esophagectomy cases died in hospital because of sepsis on postoperative days 14, 30, and 41 and major bleeding on postoperative day 54.

The one surviving case (no. 1) was a 65-year-old man who underwent reconstruction without severe complications, and was still alive without signs of infection at 424 days postoperatively.

## Conclusions

Delayed esophageal perforation secondary to thoracic aortic replacement or thoracic endovascular aortic repair (TEVAR) is a rare but potentially fatal condition [2, 4–7].

Seto et al. reported that although the exact mechanism of secondary esophageal perforation after stent grafting remains unknown, hypotheses include (1) direct erosion of the stent graft into the esophagus, (2) pressure necrosis caused by the self-expanding endoprosthesis, (3) ischemic esophageal necrosis due to disruption of the arteries that feed the esophagus, (4) infection of the stent-graft prosthesis (artificial graft for aortic replacement was included in our case), (5) pseudoaneurysm development, and (6) endoleakage into the residual aneurysmal sac [7].

In our cases that was performed aortic replacement procedure (nos. 1–6), (3) and (4) were thought to be a possible cause. In addition, no. 3 case had potential for (2), and no. 5 had potential for (2) and (5). No. 7 with Marfan syndrome had highly potential for (5) and (6). There was no equivalent case for (1).

We consider the hypothesis that ischemic esophageal necrosis due to disruption of the arteries that feed the esophagus should be focused. The thoracic esophagus is fed by bronchial and esophageal branches of the thoracic aorta. Aortic replacement or stent grafting can potentially damage these feeding arteries of the thoracic esophagus. We consider that the relatively long period from cardiovascular surgery to esophageal perforation supports this hypothesis. Uncontrolled continuous localized infection including artificial graft infection seems certain to aggravate esophageal wall ischemia and disruption in a similar way.

Eggebrecht et al. reported that they observed mild erosive lesions in the esophagus that led to perforation on endoscopy [4]. This suggested that the lesion took some time to progress to perforation, and ischemic change of the esophageal wall occurred gradually. They also mentioned that recognition of this pre-perforation state could have prompted early triage and/or surgical repair before esophageal perforation.

In no. 5 case, we had recognized redness and erosive change of the esophagus on endoscopy before the aortic replacement. If we had decided esophagectomy at that point, we might avoid poor prognosis of the case.

The prognosis of esophageal perforation cases after aortic replacement/stenting for thoracic aortic dissection or aneurysm is extremely poor especially in the elderly cases. In the elderly cases (over 80 years old), nos. 3, 4, and 5 died 41, 14, and 30 days with sepsis and other severe complications after the esophagectomy, respectively (Table 2.). Therefore, the indication for highly invasive esophagectomy should be decided carefully. We surgeons should restrict the esophagectomy to sustainable patients for invasive surgery in consideration of age and complications. We want to suggest elderly cases over 80 years old should be refrained from the esophagectomy.

It is important to control infection including regional infection and progression of cardiovascular disease for successful treatment as the result of a survival case (no. 1).

Artificial graft/stent with chronic infections was considered to be removed for long survival. We also consider that it is important to perform cardiovascular surgery with attention to maintaining esophageal blood flow.

Vascular-rich tissue filling (muscle flap or omental) to the infection site after esophagectomy may be useful for infection control. Our surviving case underwent intercostal muscle flap filling which could control prosthetic graft infection.
